# Using a monoplane stereoscopic videoradiography configuration to accurately measure skeletal positions: a proof-of-concept study

**DOI:** 10.1088/1873-4030/ae6cfd

**Published:** 2026-05-22

**Authors:** Tomasz Bugajski, Derek D Lichti, Payam Zandiyeh

**Affiliations:** 1 Department of Orthopedic Surgery, University of Texas Health Science Center at Houston, 7000, Fannin St, Houston, TX 77030, United States of America; 2 Department of Geomatics Engineering, Schulich School of Engineering, University of Calgary, 2500 University Dr NW, Calgary, Alberta T2N 1N4, Canada

**Keywords:** monoplane, stereoscopy, videoradiography, accuracy, calibration, kinematics

## Abstract

Biplanar videoradiography is a validated method for measuring skeletal kinematics. However, using two x-ray source-detector pairs introduces geometric constraints that can compromise image quality. The spatial configuration of the two pairs often requires a larger object-to-image distance (OID), leading to greater magnification and geometric blur. Consequently, increased magnification can limit the effective field of view, restricting multi-joint analyses. Additionally, geometric blur degrades image quality, potentially compromising subsequent bone registrations. To address this limitation, this proof-of-concept study evaluated a monoplane stereoscopic videoradiography (MSV) configuration. MSV uses two x-ray sources captured by a single detector, enabling a smaller OID. A pseudo-dynamic experiment was conducted to assess whether MSV would achieve accurate calibration and subsequent three-dimensional (3D) bone positions. A cube and pelvis phantom were rotated 360° on a turntable in 15° increments. At each increment, positions were recorded using both conventional motion capture (MOCAP) and MSV, with MOCAP acting as the reference standard. During MSV image acquisition, a lead barrier alternately blocked one x-ray source, allowing the other to project onto the detector. Calibration was performed using a bundle adjustment technique, and 3D bone positions were calculated using radiostereometric analysis and model-based tracking and compared against MOCAP. Overall, MSV achieved sub-millimeter and sub-degree accuracy. The calibration yielded a mean residual of 0.22 mm, while rotational and translational biases ranged from 0.36–1.16° and 0.22–1.23 mm, respectively, with precisions of 0.09–0.59° and 0.18–0.58 mm. These results support the feasibility of using MSV to accurately calculate skeletal positions. This approach reduces magnification and, by extension, geometric blur, opening avenues for multi-joint analyses and improved imaging of deeper joints. Future work will focus on implementing a true asynchronous MSV acquisition to extend this approach to dynamic, *in vivo* kinematic studies.

## 1. Introduction

Biplanar videoradiography (BVR), also known as dual fluoroscopy or dynamic stereo x-ray, has become a widely accepted reference standard for measuring three-dimensional (3D) skeletal kinematics for different joints across the body due to its consistent ability to achieve sub-millimeter and sub-degree accuracies (Bey *et al* 2006, 2008, Li *et al* 2008, Anderst *et al* 2009, 2011, Miranda *et al* 2011, Lin *et al* 2013, Iaquinto *et al* 2014, Sharma *et al* 2015, Wang *et al* 2015, Akhbari *et al* 2019, Pitcairn *et al* 2020). These levels of accuracy are crucial for biomechanics, as they enable the detection of subtle kinematic changes that may lead to abnormal joint loading and subsequent degeneration. BVR typically uses two synchronized x-ray sources and their respective orthogonally aligned detectors to directly record *in vivo* bone movements during muscle-driven physiological activities. During post-processing, subject-specific bone models are registered frame by frame to their x-ray image counterparts throughout the movement, which is then used to calculate joint biomechanics. Therefore, acquiring high-quality x-ray images with optimal spatial resolution at the joint of interest is paramount.

To acquire optimal images of the joint(s) of interest, the BVR components can be configured to varying inter-beam angles, and the subject strategically positioned within the field of view. Careful attention to these factors can minimize tissue attenuation and enhance the visualization of bony landmarks, which are critical for model registration. Additional determinants of image quality include the x-ray tube parameters (i.e. voltage, current, and pulse width), scatter reduction, and subject positioning (Tompe and Sargar 2025). While tube parameters can be adjusted to optimize image contrast within a given configuration, subject positioning is inherently constrained by the system’s configuration.

**Figure 1.**
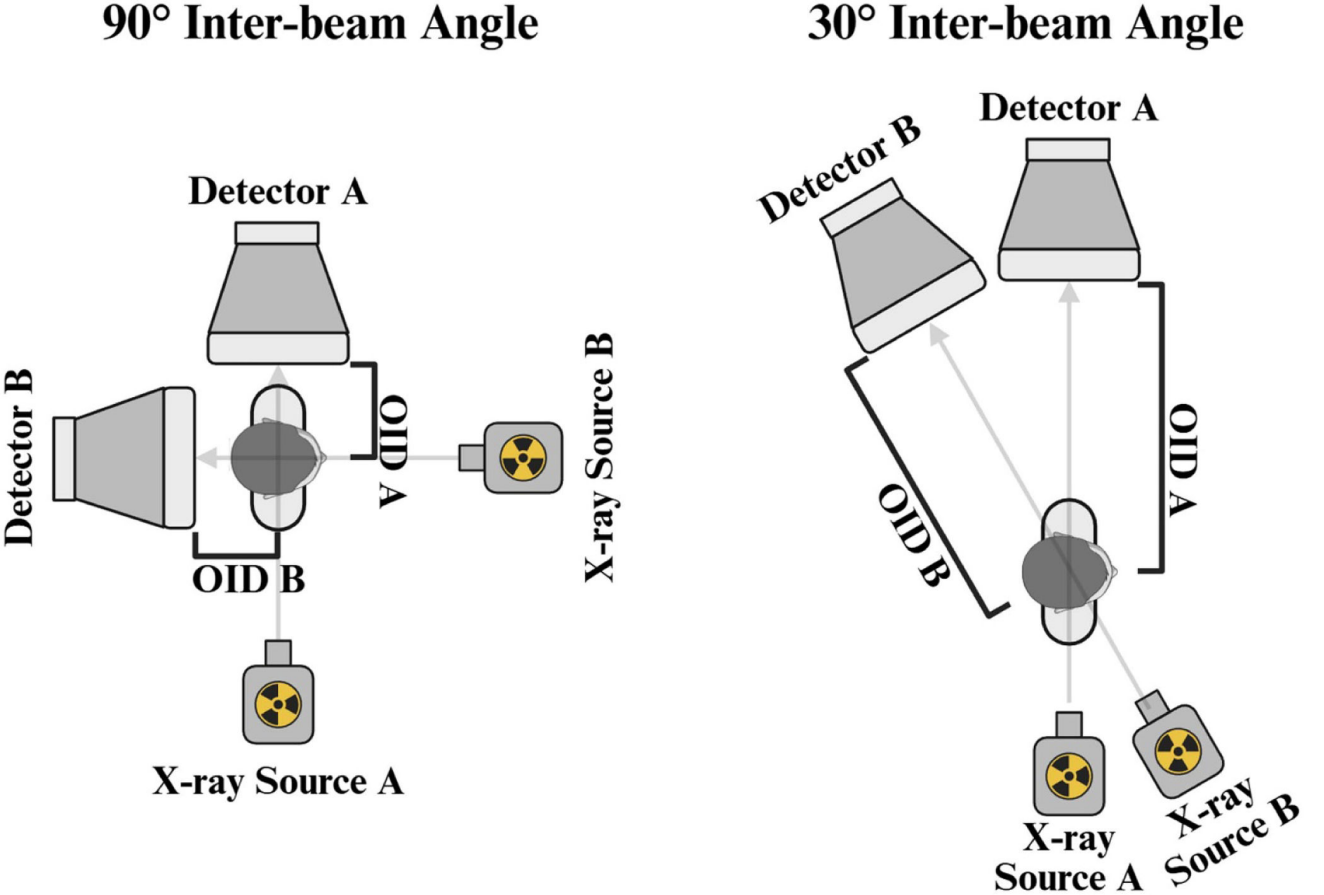
The effect of the inter-beam angle on object-to-image distance (OID). A 90° inter-beam angle results in the smallest possible OID when using biplanar videoradiography (left). However, not all bones/joints are optimally imaged using this orientation and require smaller inter-beam angles. Consequently, smaller inter-beam angles, such as 30°, result in larger OID (right). Created in BioRender. Bugajski T (2026) https://BioRender.com/pudplov.

In BVR, the participant must be positioned further away from the detectors, located at the intersection of the x-ray beams. This consideration ensures that the joint(s) of interest remain centered on both detectors throughout the range of motion. A 90° inter-beam angle provides the smallest object-to-detector distance (OID), but not all joints or activities can be imaged optimally with this arrangement. Consequently, smaller inter-beam angles (as low as 30°) are sometimes required, thereby increasing OID (figure 1).

**Figure 2.**
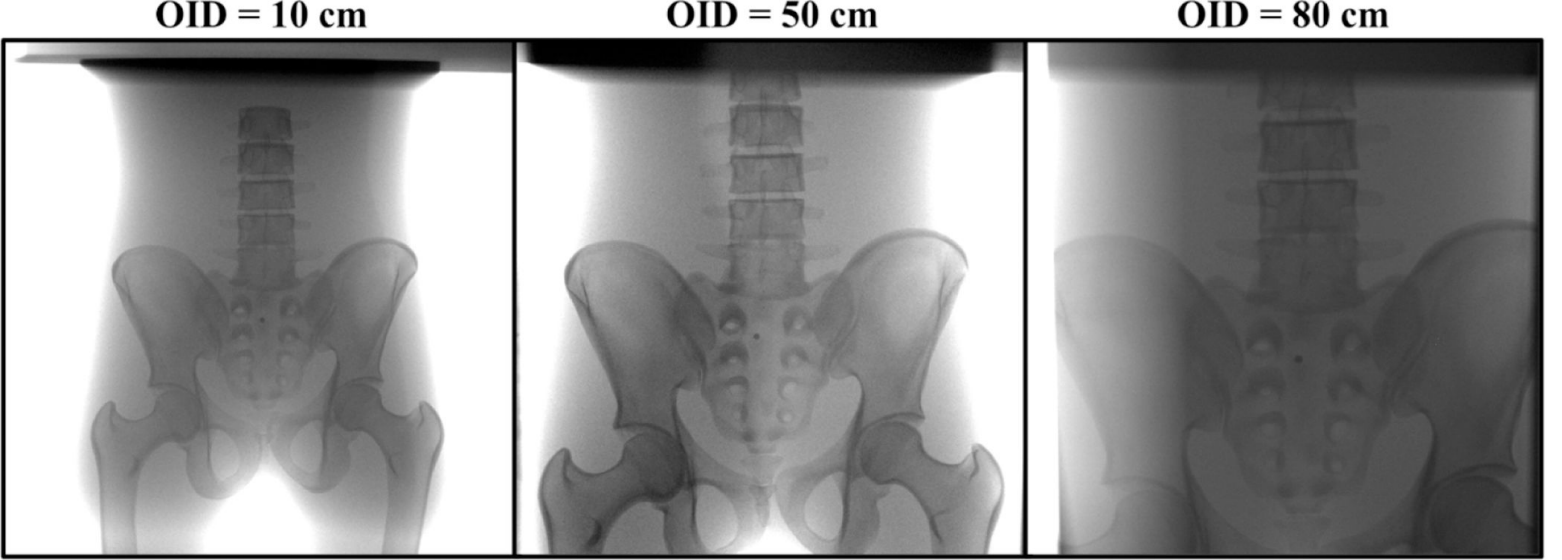
Influence of object-to-image distance (OID) on magnification. As the OID increases, bone magnification also increases, potentially obscuring surrounding structures of interest. For example, an OID of 10 cm (left image) offers the most suitable images for simultaneous assessment of lumbar spine and hip kinematics. At an OID of 50 cm (middle image, approximate OID at a 90° inter-beam angle), imaging remains acceptable; however, there is an increased risk of bones moving out of the field of view depending on the task. An OID of 80 cm (right image, approximate OID at a 30° inter-sbeam angle) significantly limits visibility, concealing the femurs and superior lumbar vertebrae, making it impractical for multi-joint analysis.

Two important and related limitations arise from having an increased OID. First, OID directly influences object magnification: a larger OID increases magnification, thereby reducing the effective field of view on the detector. This constraint can significantly limit joint analyses, often restricting imaging to a single joint. In contrast, reducing the OID expands the field of view, allowing a larger anatomical region to be captured (figure 2). This advantage may facilitate multi-joint imaging and analyses that were otherwise challenging with conventional BVR configurations.

Second, the increased magnification associated with a larger OID also leads to greater geometric blur. This blur degrades spatial resolution and may adversely affect subsequent bone registrations, especially for smaller or more complex structures such as the vertebrae. Reducing the OID mitigates geometric blur, thereby enhancing image sharpness (Sanctorum *et al* 2019).

**Figure 3.**
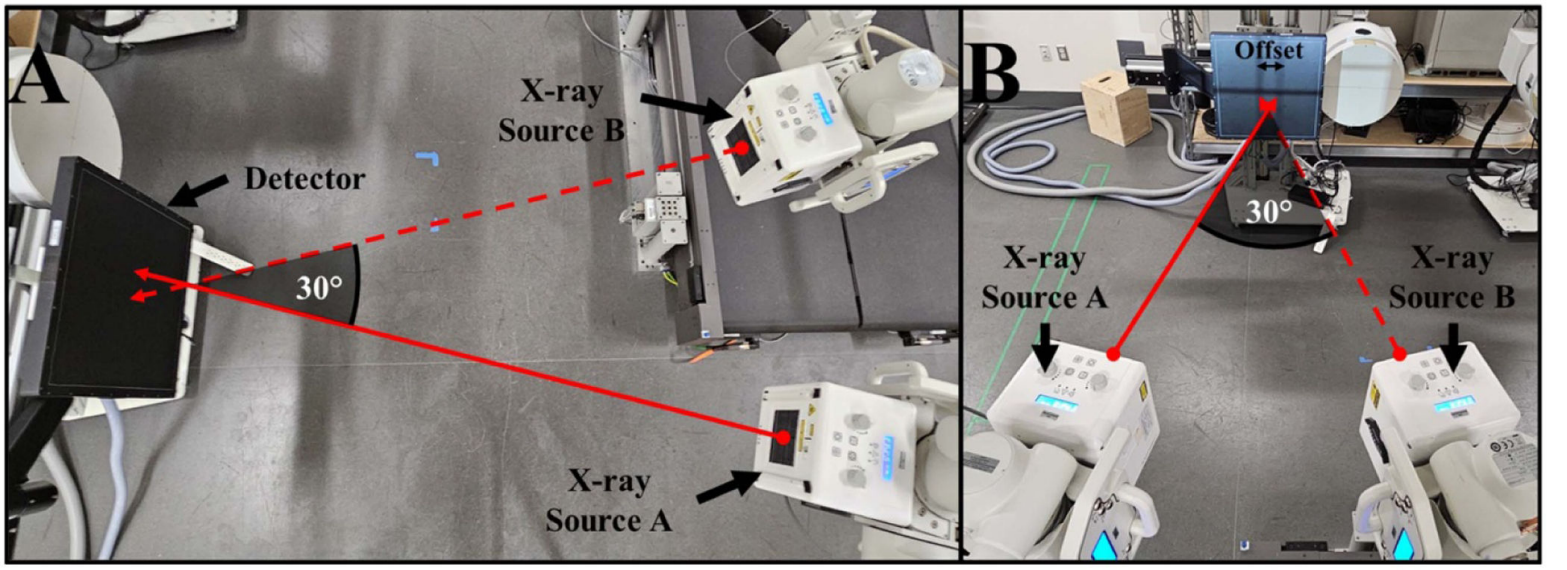
The monoplane stereoscopic videoradiography (MSV) configuration. (A) X-ray sources A and B are obliquely aligned to a single detector. This enables the participant to be positioned adjacent to the detector, minimizing the object-to-image distance. (B) X-ray sources A and B were aligned obliquely to a single detector with an inter-beam angle of 30°. The projections onto the detector were vertically centered and horizontally offset.

To overcome the limitations imposed by increased OID, this study introduces a novel imaging approach: monoplane stereoscopic videoradiography (MSV). This approach includes two asynchronous x-ray sources obliquely aligned to a single detector, allowing the participant to be positioned closer to the detector (figure 3) and enabling high-quality imaging under more flexible geometric conditions. The objectives of this proof-of-concept study were to demonstrate the feasibility of MSV for joint imaging and to quantitatively assess its accuracy and precision in (1) system calibration and (2) subsequent 3D bone positions.

## 2. Methods

### 2.1. MSV configuration

The MSV setup consisted of two x-ray sources (G-1082, Varex Imaging Corporation, USA) obliquely aligned to a single detector with a 139 *μ*m pixel pitch (Mercu 1717HS, iRay, China) (figure 3). The inter-beam angle between the sources was 30°, with a source-to-image distance of approximately 180 cm. The x-ray sources were vertically centered on the panel but horizontally offset to ensure visibility of the objects within the field of view.

**Figure 4.**
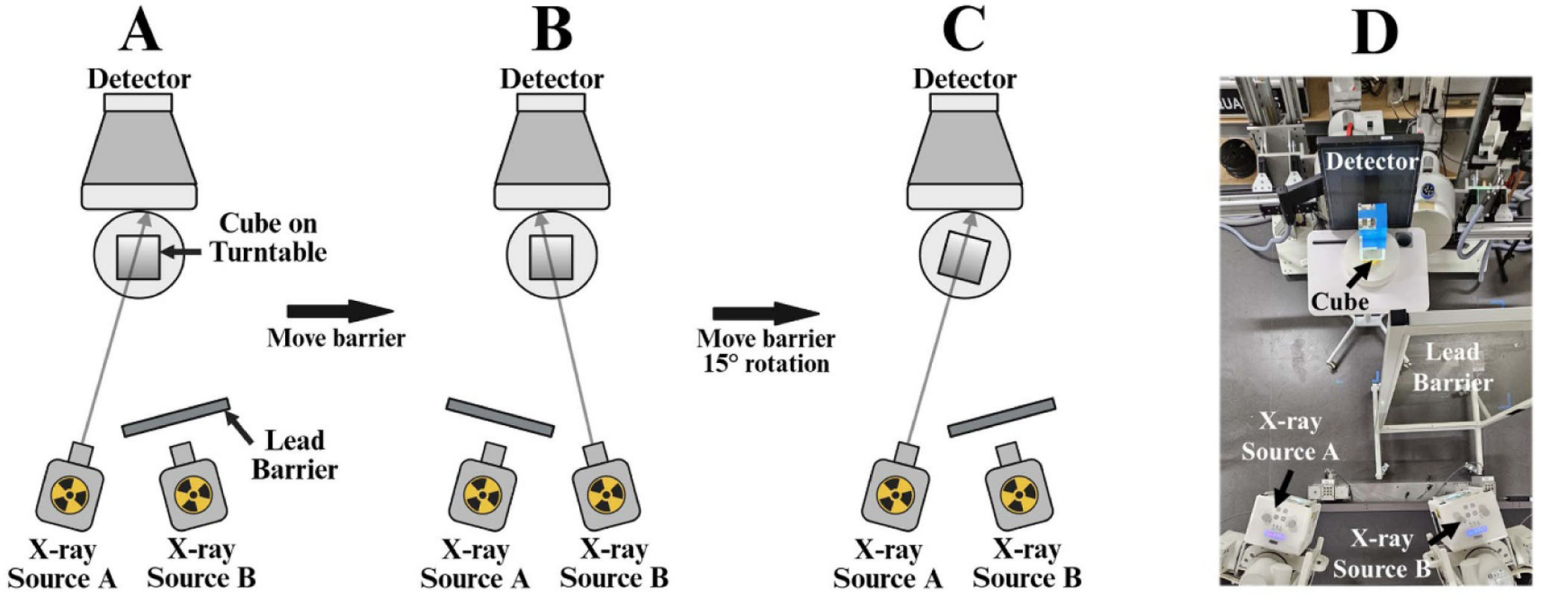
Protocol for monoplane stereoscopic videoradiography (MSV) data acquisition. (A) At the first position of the cube/phantom, the lead barrier was placed in front of x-ray source B, and images from x-ray source A were acquired in conjunction with reflective marker positions. (B) The barrier was then moved in front of x-ray source A to acquire images from x-ray source B. (C) The barrier was moved back to x-ray source B, and the cube/phantom was rotated 15° to the second position to repeat the imaging process. This process was performed until a full rotation (360°) was performed, for a total of 25 positions. (D) Image of the MSV data collection setup within the laboratory setting. Created in BioRender. Bugajski T (2025) https://BioRender.com/q22z9kc.

Given that the purpose of this study was to evaluate calibration and 3D bone position accuracy using MSV, a proof-of-concept study design was implemented. In this setup, the system continued to emit synchronous x-rays, but one source was occluded by a mobile lead barrier. By alternately shifting the barrier to obstruct one x-ray source at a time, pseudo-dynamic images were acquired from each x-ray source independently, thereby simulating the asynchronous acquisition conditions required for a true MSV approach (figure 4).

### 2.2. Data acquisition

Two pseudo-dynamic datasets were acquired using the MSV protocol described above. The first dataset involved a calibration cube embedded with tantalum beads, designed for BVR calibration (Knörlein *et al* 2016), to establish the system calibration parameters and quantify the accuracy of its 3D position using radiostereometric analysis (RSA). The second dataset involved a pelvis phantom (Model RS-113, Radiology Support Devices Inc., USA) to demonstrate MSV’s ability to image an anatomically relevant structure and to characterize the accuracy of its 3D positions using model-based registration.

Since the accuracy of the turntable rotation was unknown, motion capture (MOCAP) was collected simultaneously with MSV and served as the reference standard. This approach was justified by the well-established high accuracy of MOCAP for assessing rigid-body kinematics in the absence of soft tissue artifacts (Richards 1999), as was the case in this study. The top surface of the calibration cube and phantom were equipped with reflective markers containing tantalum beads placed within their centers. As such, each dataset consisted of (1) reflective marker trajectories acquired from a 12-camera (Oqus, Qualisys, SWE) MOCAP system and (2) x-ray images containing the cube/phantom and their respective tantalum beads. The exported x-ray images had a resolution of 1024 × 1024 px and a pixel pitch of 417 *μ*m.

#### Data collection for the calibration cube:

For the first dataset, the calibration cube was centered on a turntable (Model MT320RL40FU-B, ComXim, China) that was positioned within the field of view of the detector. Data collection was performed following the pseudo-dynamic MSV approach described earlier (x-ray tube parameters of 75 kVp, 125 mAs, and a 2 ms pulse width). After acquiring images from both views, the turntable was rotated approximately 15° using its built-in positioning feature to place the calibration cube in a second orientation. Reflective marker trajectories and x-ray images were then collected again in this new position. This process was repeated until a full 360° rotation was performed, resulting in 25 positions (0°–360° in 15° increments).

#### Data collection for the pelvic phantom:

Following data collection with the calibration cube, a second dataset was acquired by positioning the pelvic phantom on the turntable and applying a similar imaging protocol. The x-ray tube parameters during phantom imaging were 95 kVp, 160 mAs, and a 4 ms pulse width, with the positioning and data collection optimized to dynamically image the L1 and L2 segments of the lumbar spine.

### 2.3. Calibration

The MOCAP system was calibrated using the standard L-frame and wand technique. The average residual provided by qualisys track manager (v2022.1, Qualisys, SWE) was recorded.

The MSV configuration was calibrated using a self-calibrating bundle adjustment (BA) technique, an effective and versatile method for camera calibration (Luhmann *et al* 2016) that has also been successfully extended to BVR system calibration (Lichti *et al* 2015). BA was used to enhance the accuracy of the 3D reconstruction, determining the calibration parameters that best fit the geometric camera model through a non-linear, least-squares regression problem. The camera model used was the pinhole model, which assumes the collinearity condition: the x-ray point source, the 3D target point (e.g. a tantalum bead in the cube), and the target’s 2D point on the x-ray image lie on a straight line. The BA process simultaneously determined several sets of parameters, including: (1) the optimal estimates of the relative orientation parameters between the two source-detector pairs (six degrees of freedom), (2) their interior orientation parameters (principal distance and principal point), and (3) the 3D bead point coordinates. In MSV, there was only one detector; therefore, the system model consisted of additional geometric constraints that enforced the stability of the six relative orientation parameters. Specifically, the constant relative orientation of the source-detector pairs at each rotation of the calibration cube needed to be enforced. The 2D principal point was the point of intersection of the detector plane’s normal vector passing through the x-ray source, and the principal distance was the length of the normal vector. Together, these parameters defined the internal geometry of each source-detector pair in the MSV system.

### 2.4. CT imaging and segmentation

A high-resolution CT scan was acquired of the phantom with a voxel size of 0.9 × 0.9 × 0.6 mm. The first and second lumbar vertebrae (L1 and L2) were segmented in 3D Slicer (stable release v5.8.1) (Fedorov *et al* 2012) using the total segmentator extension (Wasserthal *et al* 2023) and the segment editor module. Models of the L1 and L2 vertebrae were created using the pre-processing module of slicerautoscoper^m^ (SAM) (Morton *et al* 2025).

### 2.5. Tracking

**Figure 5.**
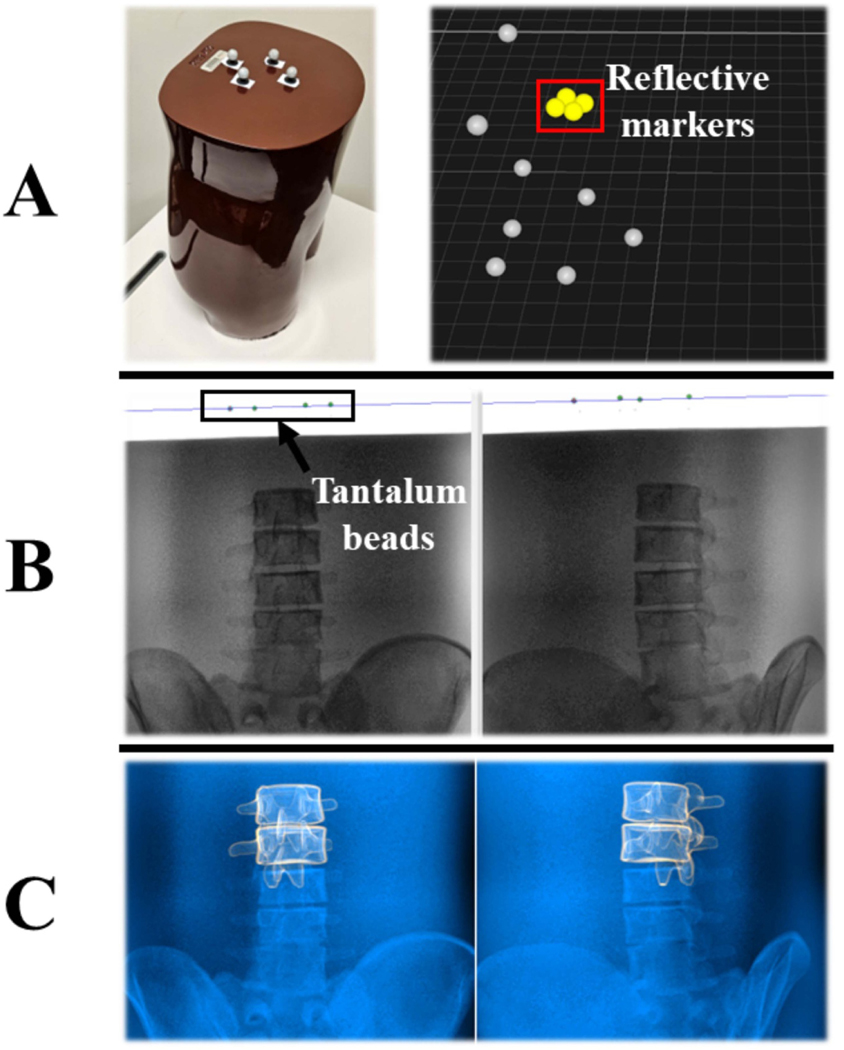
The three methods that were used to calculate the orientations of the cube and phantom (cube not shown). (A) Reflective markers were placed on the object and tracked, providing 3D positions of the markers. (B) Tantalum beads embedded within the reflective markers were tracked using radiostereometric analysis. (C) 3D/2D registrations were performed for lumbar vertebrae L1/L2 (specific to the phantom).

Three methods were used to calculate the 3D positions of the cube and phantom (figure 5):

1. **MOCAP (reference standard):** Reflective marker locations were tracked to provide accurate positions in the absence of skin motion artifacts.
2. **RSA:** Tantalum bead locations embedded in the reflective markers were tracked using RSA. This method is typically considered a reference standard in BVR validation studies.
3. **Model-based (3D/2D registration):** Tracking the L1 and L2 vertebral positions using model-based 3D/2D registration techniques applied to the monoplane x-ray images.

The 3D locations of the reflective markers were acquired within the MOCAP coordinate system by labeling them in qualisys track manager (Qualisys, SWE). Similarly, 3D locations of the tantalum beads within the MSV coordinate system were acquired using XMALab (Knörlein *et al* 2016). Specifically, the MSV calibration parameters were imported into the software, enabling the identification of the 2D bead centroids in each x-ray view and subsequent transformation into MSV space. Finally, the rotation matrices of the L1 and L2 bone models were acquired through a 3D/2D registration process in SAM. The registration process was performed within the MSV coordinate system.

Because the 3D trajectories of the reflective markers were within MOCAP space, a transformation between MOCAP and MSV was calculated to ensure all data were reported within the same coordinate system (i.e., the MSV space). The transformation between MOCAP and MSV was obtained by using the ‘estgeotform3d’ function in MATLAB (R2023b, MathWorks Inc., USA), using the 3D locations of the reflective markers (MOCAP space) and their embedded tantalum beads (MSV space) as the inputs. This transformation was then applied to the reflective marker locations, placing them in MSV space.

### 2.6. 3D bone positions

Custom MATLAB code was used to calculate the pseudo-dynamic positions of the cube and phantom. First, the rotation matrices from the first/initial position (0°) to the incremental positions (15°, 30°, …, 360°) were calculated (defined as $R_{1}^{i}$, where i=[1,25]). For the reflective marker and tantalum bead locations, $R_{1}^{i}$ was obtained using the same ‘estgeotform3d’ function mentioned previously. For the L1 and L2 bone models, $R_{1}^{i}$ was calculated using equation (1):

(1) $$R_{1}^{i}=R_{O}^{i}*R_{O}^{1T}$$

where $R_{O}^{i}$ is the rotation matrix for L1/L2 at position i and $R_{O}^{1}$ is the rotation matrix for L1/L2 at the initial position.

To compare the methods, another rotation matrix was calculated between MOCAP (the reference standard) and the other two methods (RSA and model-based) using equation (2):

(2) $$R_{nM1}^{i}=R_{n1}^{i}*R_{M1}^{iT}$$

where $R_{nM1}^{i}$ is the relative rotation matrix between MOCAP $\left(R_{M1}^{i}\right)$ and RSA or model-based $\left(R_{n1}^{i}\right)$. The relative rotation matrices between each method were then used to calculate their differences in 3D rotations and translations. Rotations were calculated using a $\left[Z,Y^{\prime },X^{\prime \prime }\right]$ Cardan angle sequence, where the Z-axis corresponded to the primary axis of rotation about the turntable. Additionally, a helical axis representation was utilized due to its common application in biomechanical analyses, especially of the spine.

The cube contained outcome measures between MOCAP-RSA, while the phantom contained measures between MOCAP-RSA, MOCAP-L1, and MOCAP-L2. For the translations, only MOCAP-RSA was suitable for comparison, as the RSA beads were embedded within the reflective markers.

### 2.7. Analysis

The quality of the calibration was assessed by calculating the root-mean-square (RMS) of the 2D residuals, defined as the difference between the bead locations seen in the x-ray images and their locations projected by the camera model. Additionally, the precision of the interior orientation parameters for each source-detector pair was represented by their standard deviations (SDs), determined through the propagation of variance (Mikhail 1982). As the precisions are inherently influenced by the geometry of the system, the precisions of the principal point and principal distance were normalized with respect to the magnitudes of these parameters.

To evaluate the accuracy of the 3D bone positions using MSV (RSA and model-based), their absolute differences to MOCAP were averaged across the full rotation (i.e. the 25 positions). Accuracy was reported as the mean values with corresponding SDs, reflecting the MSV configuration’s bias and precision, respectively.

## 3. Results

### 3.1. Calibration

As expected, the MOCAP calibration achieved submillimeter accuracy, with a bias of 0.88 mm and precision of 0.35 mm.

The accuracy of the MSV calibration parameters is summarized in table 1. A sub-millimeter model fit was achieved between the x-ray image bead locations and their respective camera model projections, with a residual RMS of 0.22 mm (0.52 px). The precisions of the (x,y) principal point coordinates were (1.42 mm [3.41 px], 0.65 mm [1.57 px]) and (1.63 mm [3.92 px], 0.66 mm [1.59 px]) for the two source-detector pairs, indicating better precision along the vertical (y) coordinate. Normalized to the detector size, these precisions were (0.33%, 0.15%) and (0.38%, 0.16%). The principal distance of the configuration was 175 cm (4200 px), with precisions of 3.47 mm (8.32 px) and 3.42 mm (8.20 px) for the two source-detector pairs, corresponding to high normalized precisions of 0.20% for both.

### 3.2. Kinematics

The 3D bone positions derived from the MSV configuration achieved sub-degree and sub-millimeter accuracy. Better accuracy was observed using Cardan angles (bias: 0.11–0.75°; precision: 0.09–0.58°) compared to the helical rotation (bias: 0.36–1.16°; precision: 0.18–0.59°), noted by the overall smaller magnitudes of bias and precision (table 2). The only rotational measure to exceed 1° was the bias of L1 (1.16°). All *XYZ* translations showed sub-millimeter bias (0.22–0.72 mm) and precision (0.18–0.58 mm) (table 3). However, the bias for the magnitudes of these translations exceeded 1 mm (1.06–1.23 mm), but the precision remained similar (0.52–0.53 mm). Among the methods, RSA demonstrated superior accuracy and precision (table 2).

## 4. Discussion

This proof-of-concept study evaluated the accuracy of a novel MSV configuration to assess its feasibility for measuring 3D bone positions. To our knowledge, this is the first implementation of this unique setup. Overall, the configuration achieved accurate calibration and pseudo-dynamic bone positions when using standard post-processing methods, with biases and precisions mostly within sub-degree and submillimeter ranges. These results support the validity of the MSV approach and provide strong motivation for investigating its utility in dynamic biomechanical assessments.

The calibration of the MSV configuration achieved a sub-millimeter model fit across both source-detector pairs, indicating high accuracy (table 1). The precision of the principal point coordinates showed more than twofold disparity (Kenefick *et al* 1972) between the *x* and *y* directions. This discrepancy arises from the functional dependence of the principal point and the camera orientation angles. Although more homogeneous precision could be achieved by adding calibration images with cube rotations about its *X* or *Y* axis (Fraser 1984), doing so would increase the complexity of the data collection process and was outside the scope of this study. Similarly, the lower precisions observed for the principal distance were expected, given the narrow field of view of the configuration (i.e. a long principal distance of 175 cm [4200 px] relative to the 43 × 43 cm [1024 × 1024 px] image format, resulting in a 13.80° field of view). In extreme cases where the field of view is below 10°, precision can degrade considerably as the x-ray beams approach a near-parallel projection, diminishing the performance of the central perspective, collinearity model (Stamatopoulos and Fraser 2011).

Importantly, although some calibration outcomes may appear large in absolute terms, they should be interpreted within the context of the system’s geometry and calibration scale. When considered relative to the imaging configuration, the observed precisions fall well within acceptable limits for accurate calibration. Notably, when normalized, the interior orientation parameters demonstrated exceptionally high precision, highlighting the effectiveness of the 360° calibration network in reducing interdependence between the principal distance and camera position parameters (Lichti *et al* 2015).

In agreement with the high calibration accuracy, the resulting bone positions substantiated the validity of the MSV configuration, with biases between 0.11–1.16° and 0.22–1.23 mm, and precisions between 0.09–0.59° and 0.18–0.58 mm (tables 2 and 3). Although direct comparisons with prior BVR validations are not possible due to the pseudo-dynamic design of the current study, the observed values fall within the ranges previously reported for the traditional BVR approach.

In one cadaveric study, BVR was validated for wrist kinematics using bone-affixed reflective markers as the reference standard (Akhbari *et al* 2019). Model-based tracking of the third metacarpal and radius achieved a bias of up to 0.50° and 0.50 mm, and precision of up to 1.1° and 0.8 mm. Several other studies have validated BVR for other joints (e.g. cervical spine (Anderst *et al* 2011), glenohumeral (Bey *et al* 2006), hip (Lin *et al* 2013), tibiofemoral (Li *et al* 2008, Anderst *et al* 2009), patellofemoral (Bey *et al* 2008), and ankle (Wang *et al* 2015, Pitcairn *et al* 2020)) using RSA as the reference standard, with the maximum bias reaching magnitudes of 1.75° and 1.54 mm, and precisions reaching magnitudes of up to 2.2° and 1.13 mm.

Another study used an ‘RSA wand’, similar to those typically used for calibrating MOCAP systems, to assess the accuracy of BVR (Iaquinto *et al* 2014). That study reported bias of 0.08° and 0.13 mm, with the corresponding precision of 0.07° and 0.12 mm (Iaquinto *et al* 2014). Lastly, two studies used highly precise instruments to simulate bone movements within the BVR system and compared the RSA and model-based tracking. Across both studies, the maximum bias was 0.44° and 0.33 mm, while the maximum precision was 0.26° and 0.30 mm. As expected, RSA consistently demonstrated superior accuracy over model-based methods, a similar finding to this study.

Overall, the bias and precision of the bone positions obtained with MSV fell within the ranges reported in comprehensive BVR studies, demonstrating its strong potential to acquire dynamic skeletal kinematics.

Given the widespread use of the helical axis, this study examined how validity outcomes are affected by angle calculation methods to aid in clearer interpretation across studies. In this regard, the helical rotation consistently demonstrated lower accuracy than the Cardan angles, particularly for the *Z*-axis rotation. This was expected, as the helical rotation considers an overall rotation about a single axis rather than three sequential, independent rotations. Due to the bias present in the secondary axes (*X* and *Y*), the helical rotation errors were compounded, resulting in larger discrepancies. The magnitude of the translations exhibited poorer accuracy than their vector components due to the same compounding effect. Consequently, the only cases where overall accuracy exceeded 1° or 1 mm were the helical rotation bias of L1 and the translation magnitudes of the cube and phantom. Nonetheless, the accuracies of the XYZ components of both rotations and translations support a high level of confidence in the MSV configuration.

This study had notable limitations. First, dynamic MSV acquisition was not performed due to hardware constraints, specifically the requirement for asynchronous x-ray emission onto a single detector, which would necessitate modifications to the current BVR system. Also, true MSV data acquisition would require advanced image interpolation techniques (Fang *et al* 2024) to post-process the asynchronous data. The accuracy of these interpolation methods would inherently depend on skeletal motion velocity and imaging rate. However, as a proof-of-concept study, the focus was on validating system calibration and data processing using a pseudo-dynamic study design. With this foundation established, future work will aim to implement fully dynamic, asynchronous x-ray acquisition and further develop post-processing methods for MSV. Additional validation studies will be necessary to determine whether comparable accuracies to those reported here and in prior BVR studies can be achieved, and to quantify error as a function of motion speed and imaging rate.

Furthermore, the current study employed a symmetrical inter-beam angle of 30° (i.e. 15° source-to-detector angles relative to the detector norm). Although this configuration demonstrated excellent reconstruction accuracy, alternative geometries, including larger or asymmetrical beam angles, were not investigated. As a sub-analysis, simulations were conducted to assess the effect of varying inter-beam angles (see supplementary document). As expected, increasing the inter-beam angle, and thus the degree of parallax, improved the precision of bead localization (maximum SD: 0.98 mm at a 15° inter-beam angle; 0.13 mm at 90° inter-beam angle). However, an empirical assessment of these configurations is warranted to establish geometric limits. While greater parallax can enhance depth discrimination, it may also introduce greater perspective distortions, modify triangulation conditioning, and alter projection geometry (Fraser 1984). These factors could ultimately influence subsequent bone registrations, reconstruction accuracy, and measurement reliability.

Another limitation of the present study was the use of MOCAP as the reference standard, which constrained the interpretation of absolute accuracy. A more advanced turntable system equipped with a high-resolution rotary encoder may provide a more rigorous ground-truth measure in future experiments. Nevertheless, in the absence of skin motion, MOCAP systems are well established as highly accurate tools for quantifying rigid-body positions, as evidenced by the reported submillimeter calibration accuracy in this study. Because this study used inanimate, rigid objects to evaluate system accuracy, the selected reference standard was considered appropriate given the investigation’s scope and proof-of-concept nature.

In relation to the previous limitation, this study was also unable to place reflective markers or tantalum beads directly on the phantom’s vertebrae, which prevented a direct comparison of translational accuracy between model-based tracking and the other methods (MOCAP and RSA). Nevertheless, translational accuracy generally mirrors the patterns observed for rotational accuracy in this study and previous BVR studies, with model-based methods exhibiting slightly lower accuracy than RSA. Therefore, although the evaluation of translational accuracy was not possible, it is unlikely to affect the overall interpretation of the findings or the assessment of MSV performance.

## 5. Conclusion

The results of this proof-of-concept study demonstrate that MSV can be used to quantify bone positions with sub-millimeter and sub-degree accuracy, comparable to established BVR systems. By reducing OID, MSV can minimize magnification and improve spatial resolution. These advantages are particularly valuable for imaging smaller and more complex anatomy, such as the vertebrae, and could enable multi-joint analyses that were previously unachievable. As a result, MSV may allow for more confident and accurate assessment of complex joint kinematics. Future work will focus on implementing true asynchronous MSV acquisition and developing advanced post-processing methods to further validate and extend this approach to dynamic, *in vivo* studies.

## Supplementary Material

Sup 1

Supplementary material for this article is available online

## Figures and Tables

**Table 1.** Accuracy of the MSV calibration parameters, specifically the model fit and the precision of the interior orientation parameters for each source-detector pair.

		Interior orientation parameters (pair 1, pair 2)		
		Principal point		
	Model fit (RMS)	*x* (horizontal)	*y* (vertical)	Principal distance

Absolute	0.22 mm (0.52 px)	1.42 mm [3.41 px], 1.63 mm [3.92 px]	0.65 mm [1.57 px], 0.66 mm [1.59 px]	3.47 mm (8.32 px), 3.42 mm (8.20 px)
Normalized	—	0.33%, 0.38%	0.15%, 0.16%	0.20%, 0.20%

**Table 2.** The rotational accuracy of the MSV configuration. Values are the bias ± precision, for each method relative to MOCAP.

Object	Tracking method	Helical (°)	Cardan (°)		
			*X*	*Y*	*Z*

Cube	RSA	0.36 ± 0.18	0.14 ± 0.11	0.27 ± 0.19	0.11 ± 0.09
	RSA	0.55 ± 0.24	0.37 ± 0.21	0.26 ± 0.20	0.15 ± 0.13

Phantom	L1	1.16 ± 0.59	0.64 ± 0.58	0.75 ± 0.44	0.41 ± 0.28
	L2	0.91 ± 0.52	0.66 ± 0.47	0.35 ± 0.28	0.40 ± 0.30

**Table 3.** The translational accuracy of the MSV configuration. Values are the bias ± precision of the RSA method relative to MOCAP.

Object	Translation (mm)			
	*X*	*Y*	*Z*	Magnitude

Cube	0.67 ± 0.43	0.72 ± 0.43	0.22 ± 0.18	1.06 ± 0.53
Phantom	0.60 ± 0.41	0.71 ± 0.58	0.53 ± 0.34	1.23 ± 0.52

## Data Availability

The data cannot be made publicly available upon publication because the cost of preparing, depositing and hosting the data would be prohibitive within the terms of this research project. The data that support the findings of this study are available upon reasonable request from the authors. Supplementary data 1 available at https://doi.org/10.1088/1873-4030/ae6cfd/data1.
