# Supplementary material for: Using a monoplane stereoscopic videoradiography configuration to accurately measure skeletal positions: a proof-of-concept study
Source: Med Eng Phys. Author manuscript; Available in PMC 2026 Aug 11. (PMC13459624; doi:10.1088/1873-4030/ae6cfd)
Supplement: Supplementary file 1 [file NIHMS2186194-supplement-Sup_1.docx]

**Supplementary Methods: Preanalysis Simulation of Localization Precision**

**Methods:** A preanalysis simulation was conducted to quantify the expected localization precision of 64 target points under varying imaging geometries. The simulation employed the variance–covariance propagation method to model how observational uncertainty influences reconstructed three-dimensional (3D) point estimates through bundle adjustment. The imaging network geometry was systematically varied by adjusting the inter-beam angle between imaging rays. Six configurations were evaluated, spanning inter-beam angles from 15° to 90° in 15° increments along the X–Y plane (**Figure S1**), representing acquisition geometries from narrow- to wide-angle with increasing geometric constraint.

**Figure S1. The six geometries used in the simulation.** Inter-beam angles were increased from 15° to 90° in 15° increments within the X–Y plane (parallel to the floor). For each angle, the standoff distance was adjusted to ensure all targets were visible in both images. The red triangles denote the two X-ray sources. As the inter-beam angle increased, the separation between the sources expanded along the X direction, while the Y direction corresponded to depth.

The spatial uncertainty associated with each reconstructed target point was visualized using 95% confidence ellipsoids, derived from the covariance matrices. The dimensions of each ellipsoid reflect the magnitude of uncertainty along each axis, where larger ellipsoids correspond to greater localization uncertainty and reduced precision, whereas smaller ellipsoids indicate more precise estimates of target position. *To improve visibility, all ellipses have been uniformly scaled by a factor of ten in the results section.*

Across all 64 target points, summary metrics were computed for each inter-beam configuration to characterize overall localization performance. Specifically, the minimum and maximum standard deviations along each spatial axis were calculated, along with the root-mean-square error (RMSE) across all beads. These metrics provide a compact quantitative summary of both the best- and worst-case localization precision within the field, as well as the aggregate reconstruction error. All measurements were reported in millimeters.

**Results: Figure S2** summarizes the simulation results. As expected, localization precision along the Y-axis (depth) improved monotonically with increasing inter-beam angle, demonstrating a strong dependence on imaging geometry. At the narrowest inter-beam angle (15°), uncertainty was highest, with standard deviations ranging from 0.86 to 0.98 mm and an RMSE of 0.92 mm. As the angle increased to 30°, Y-axis precision improved substantially (SD: 0.42–0.48 mm; RMSE: 0.45 mm), followed by continued reductions at 45° (SD: 0.27–0.32 mm; RMSE: 0.29 mm). Further gains were observed at 60° (SD: 0.19–0.23 mm; RMSE: 0.21 mm). At 75° and 90°, the highest precision was achieved, with SDs decreasing to 0.14–0.17 mm (RMSE: 0.16 mm) and 0.11–0.13 mm (RMSE: 0.12 mm), respectively. Overall, this represents an approximately eightfold reduction in RMSE from 15° to 90˚.

In contrast, localization precision along the X and Z axes remained invariant with respect to inter-beam angle, with standard deviations consistently ranging from 0.23 to 0.25 mm and an RMSE of 0.24 mm across all geometries.

|   Y: Min 0.86 \| Max 0.98 \| RMSE 0.92 |   Y: Min 0.42 \| Max 0.48 \| RMSE 0.45 |   Y: Min 0.27 \| Max 0.32 \| RMSE 0.29 |
| --- | --- | --- |
|   Y: Min 0.19 \| Max 0.23 \| RMSE 0.21 |   Y: Min 0.14 \| Max 0.17 \| RMSE 0.16 |   Y: Min 0.11 \| Max 0.13 \| RMSE 0.12 |

**Figure S2. Effect of inter-beam angle on localization precision.** Simulated 95% confidence regions for target localization are shown for each inter-beam angle. Ellipses represent the uncertainty in the X–Y plane (Z axis not shown) and are *scaled by a factor of 10 for visualization*. Each panel displays the spatial distribution of the 64 target points. As the inter-beam angle increased, a marked reduction in uncertainty was observed, specifically along the Y-axis (depth), reflecting improved parallax and geometric constraint in the imaging system.

**Conclusions:** The simulation results demonstrate that localization precision is strongly influenced by imaging geometry, with increasing inter-beam angle improving depth (Y-axis) precision through enhanced parallax. Accordingly, variation in inter-beam angle primarily affected localization along this axis.

The observed anisotropy in localization uncertainty reflects the directional nature of triangulation, in which depth estimation is more sensitive to inter-beam angle than lateral (X-axis) position. At small inter-beam angles, limited parallax leads to poor depth resolution and elongated uncertainty ellipsoids along the Y-axis, whereas larger angles provide stronger geometric constraint and more isotropic uncertainty. These findings highlight the direction-dependent nature of uncertainty propagation under controlled geometric conditions.

Several limitations should be considered. The simulation assumes idealized conditions, including fixed noise characteristics and simplified geometry, and may not fully capture real-world sources of error such as calibration inaccuracies, detector nonuniformity, or environmental variability. Additionally, geometric variation was applied within a single plane, and thus directional effects were not fully evaluated in 3D; consequently, the observed improvements are specific to the tested configuration.
